# Cascaded counter-propagating nonlinear interactions in highly-efficient sub-µm periodically poled crystals

**DOI:** 10.1038/s41598-017-07016-y

**Published:** 2017-08-14

**Authors:** Andrius Zukauskas, Anne-Lise Viotti, Charlotte Liljestrand, Valdas Pasiskevicius, Carlota Canalias

**Affiliations:** Department of Applied Physics, Royal Institute of Technology, Roslagstullsbacken 21, 10691 Stockholm, Sweden

## Abstract

Mirrorless optical parametric oscillators (MOPOs) are very attractive parametric devices that rely on the nonlinear interaction of counter-propagating photons to inherently establish distributed feedback, without the use of external mirrors or surface coatings. These devices offer unique spectral and coherence properties that will benefit a large variety of applications ranging from spectroscopy to quantum communications. The major obstacle in exploiting their full potential is ascribed to the difficulty in engineering a nonlinear material in which the generation of counter-propagating waves can be phase matched. Here we present a reliable and consistent technique for fabrication of highly-efficient sub-micrometer periodically poled Rb-doped KTiOPO_4_. We experimentally demonstrate the first cascaded counter-propagating interactions in which the generated forward signal serves as a pump for a secondary MOPO process, reaching pump depletion larger than 60%. The cascaded process exemplifies the high efficiency of our nonlinear photonic structures. Our domain-engineering technique paves the way to realize counter-propagating schemes and devices that have been deemed unfeasible until now.

## Introduction

Mirrorless optical parametric oscillators (MOPOs) are a special class of optical parametric oscillators based on the three-wave mixing process in which the pump photon is split into two photons, signal and idler, propagating in opposite directions^1^. MOPOs do not rely on reflected waves or a cavity to establish the positive feedback for oscillation. Instead, the distributed feedback is automatically accomplished by the nonlinear interaction of counter-propagating photons. MOPO is a very simple, self-aligned^2^ parametric oscillator that does not require any additional components, making it a compact and robust light source. The photon momentum conservation condition in counter-propagating three-wave mixing puts strong constraints on the phase-matching scheme and renders the unique spectral, tuning and coherence properties of MOPO^3^. At the same time, satisfying the momentum conservation represents the major difficulty in practically realizing the counter-propagating parametric interactions in the visible, near-infrared and mid-infrared spectral ranges. Since birefringent phase-matching in this case demands an unnaturally large material birefringence^4^, quasi-phase matching (QPM)^5^ becomes the most viable route to fulfill the phase-matching condition. However, the periodicity of the QPM structure has to be of the order of half of the wavelength of the backward propagating wave, ~*λ*/2*n*, where *n* is the index of refraction. Therefore, even in oxide ferroelectrics with relatively low index of refraction, such as KTiOPO_4_ (KTP) and LiNbO_3_, which are commonly used in QPM devices, the structures for counter-propagating interactions in the near- and mid-infrared spectral ranges must have sub-micrometer periodicity for first-order QPM. In nonlinear semiconductors, such as GaAs, the required periodicity would be approximately two-times smaller owing to larger index of refraction. Fabrication of periodic structures with such short periods presents a modern-day engineering challenge. Indeed, MOPO had to wait 41 years to go from a theoretical possibility^1^ to its first experimental demonstration^6^. Low-threshold and energy-scalable MOPO requires an interaction length of the order of a centimeter in structures that are homogeneous over a large aperture. Unfortunately, the standard structuring techniques used in ferroelectric nonlinear crystals cannot be relied upon to produce consistently high-quality sub-µm-periodicity QPM crystals required for low threshold MOPO, which explains the long delay until the first experimental demonstration.

One of the salient achievements presented in this work is the development of the novel technique which allows consistent fabrication of high quality sub-micrometer-periodicity QPM structures in bulk Rb-doped KTiOPO_4_ (RKTP) crystals. Up to now, MOPO has only been demonstrated in sub-µm periodically poled KTP crystals of limited aperture (0.4 mm) and length (5 mm). Although multiple efforts have been made to fabricate sub-µm periodically poled crystals (see for instance, ref. 7 and references within), the available methods still lack reproducibility and the capability to obtain large apertures and highly-efficient gratings with long interaction lengths. Here, we present a reliable and consistent method for fabrication of highly homogeneous bulk sub-µm domain gratings in 1 mm thick RKTP crystals. This technique will be the key to realization of new classes of devices with a broad range of functionalities. Examples of these are: slow-light devices^8^; bistable oscillators and optical switches; sources of nonclassical light such as biphoton generators^9^; frequency translators allowing the transfer of the photon quantum state from one wavelength to another^10^; counter-propagating optical amplifiers with the expected self-compression of the counter-propagating pulses^11^; as well as simple and robust high-energy sources for narrowband mid-infrared radiation.

In particular, in this work we show that the high-quality 1-mm thick RKTP QPM structures fabricated using our new technique, allow for demonstration of a highly-efficient counter-propagating oscillator which exhibits rather unusual cascading behavior. Cascaded MOPO processes in the same crystal happen when the signal photons generated in the primary MOPO process are further split and pump a secondary MOPO process. Both the primary and the secondary processes would satisfy QPM momentum conservation conditions:

1 $$\begin{array}{rcl}{k}_{{\rm{p}}} & = & {K}_{{\rm{G}}}+{k}_{{\rm{s}}1}-{k}_{{\rm{i}}1},\\ {k}_{{\rm{s}}1} & = & {K}_{{\rm{G}}}+{k}_{{\rm{s}}2}-{k}_{{\rm{i}}2}.\end{array}$$

Here the subscripts *s, p* and *i* denote signal, pump and idler respectively; *1* and *2* refer to the primary and secondary processes. *K*_***G***_ = 2πm/Λ is the QPM grating vector, m is the order of the interaction (m = 1 in our case), and Λ is the period of the grating. Obviously energy conservation is automatically fulfilled for both processes, such that *ω*_*p*_ = *ω*_*s*1_ + *ω*_*i*1_, and *ω*_*s*1_ = *ω*_*s*2_ + *ω*_*i*2_.

**Figure 1:**
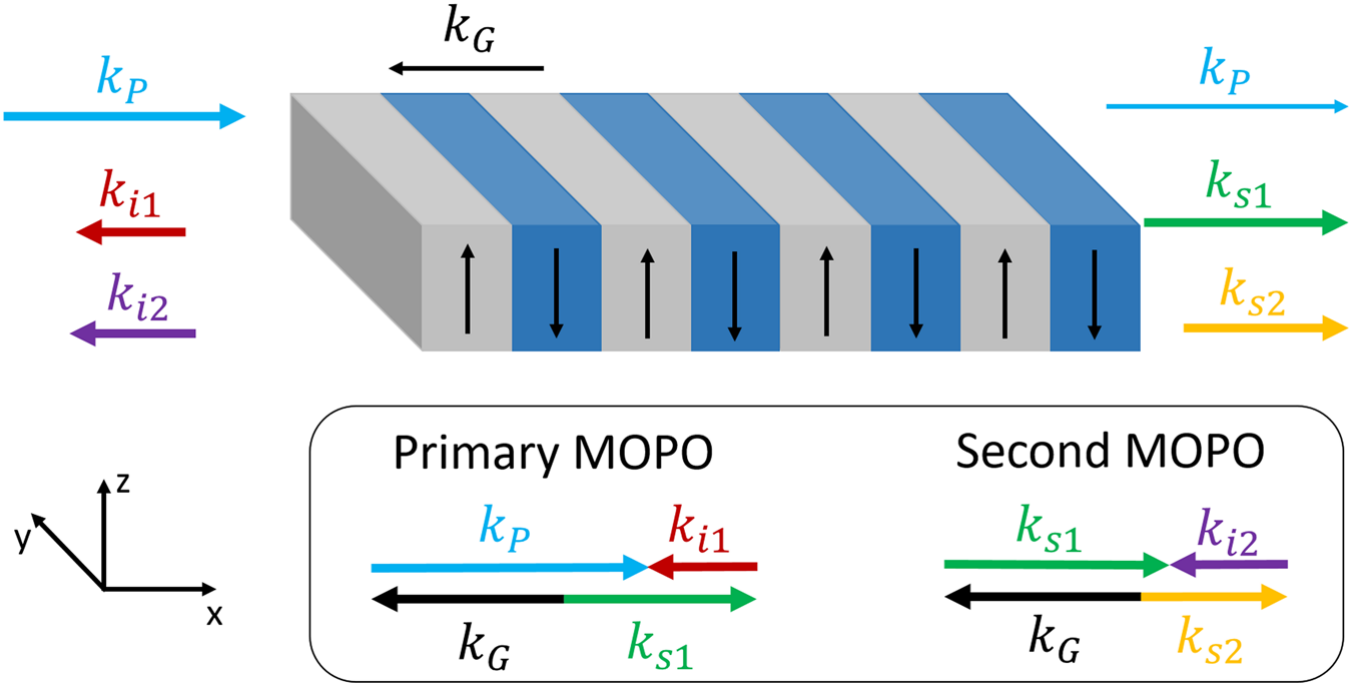
Cascaded MOPO scheme. Schematic of the cascaded counter-propagating interactions and wave-vector diagram for the primary and cascaded MOPO. Subscripts correspond to *p* - pump, *G* – QPM grating, *s1* – signal 1, *s2* – signal 2, *i1* – idler 1, *i2* – idler 2. The Cartesian axes indicate the orientation of the PPRKTP crystal axes.

**Figure 2:**
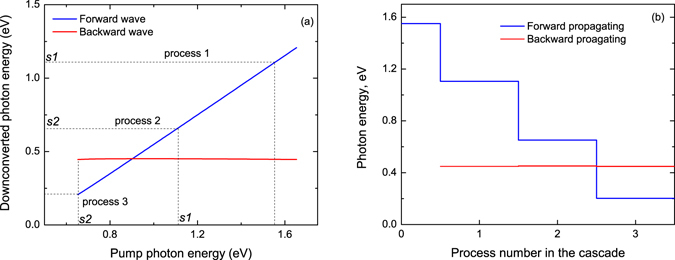
Calculated cascaded MOPO processes in a 755 nm-period PPRKTP with a primary pump-photon energy of 1.55 eV. (**a**) Cascade steps in the forward propagating photon energy as cascaded MOPO processes sequentially start oscillating. On the axes, *s1* and *s2* indicate the energy of the generated signals that are used as pumps for the consecutive cascades. Note that the energy of the backward-propagating photons remains relatively stable. (**b**) Energy levels of the generated photons in the cascaded processes.

A sketch of a cascaded MOPO with forward-travelling signals, together with the corresponding wave-vectors diagrams, is depicted in Fig. 1. In order to illustrate the cascaded MOPO behavior we calculated the energy of the forward- and backward-generated photons as the function of the pump-photon frequency in periodically-poled RKTP (PPRKTP) with a periodicity of 755 nm, as shown in Fig. 2 (a). In this calculation we used the Sellmeier expansion from ref. 12. In MOPO, the frequency of the forward-propagating wave depends, to a large extent, linearly on the frequency of the pump photon, while the frequency of the backward-generated photons changes very little in comparison. Therefore, if the QPM crystal is of high quality and the MOPO threshold is low then the cascade will happen as shown in Fig. 2(a) for the case of an initial pump-photon energy of 1.55 eV, where in each cascade step the forward propagating photon will lose a portion of the energy almost equal to the energy of the backward-propagating photon. Owing to the linear dependency of the forward-propagating photon frequency on that of the pump, any frequency modulation present in the pump will be translated through this cascade to sequentially lower frequencies. This is radically different from conventional optical parametric oscillators (OPOs) with co-propagating waves, in which phase-matched cascaded effects typically require complex cavity set-ups and multiple-grating QPM structures. With the QPM crystals used in this work, primary-pump conversion efficiencies in excess of 60% are demonstrated.

## Fabrication of sub-µm periodically poled crystals

**Figure 3:**
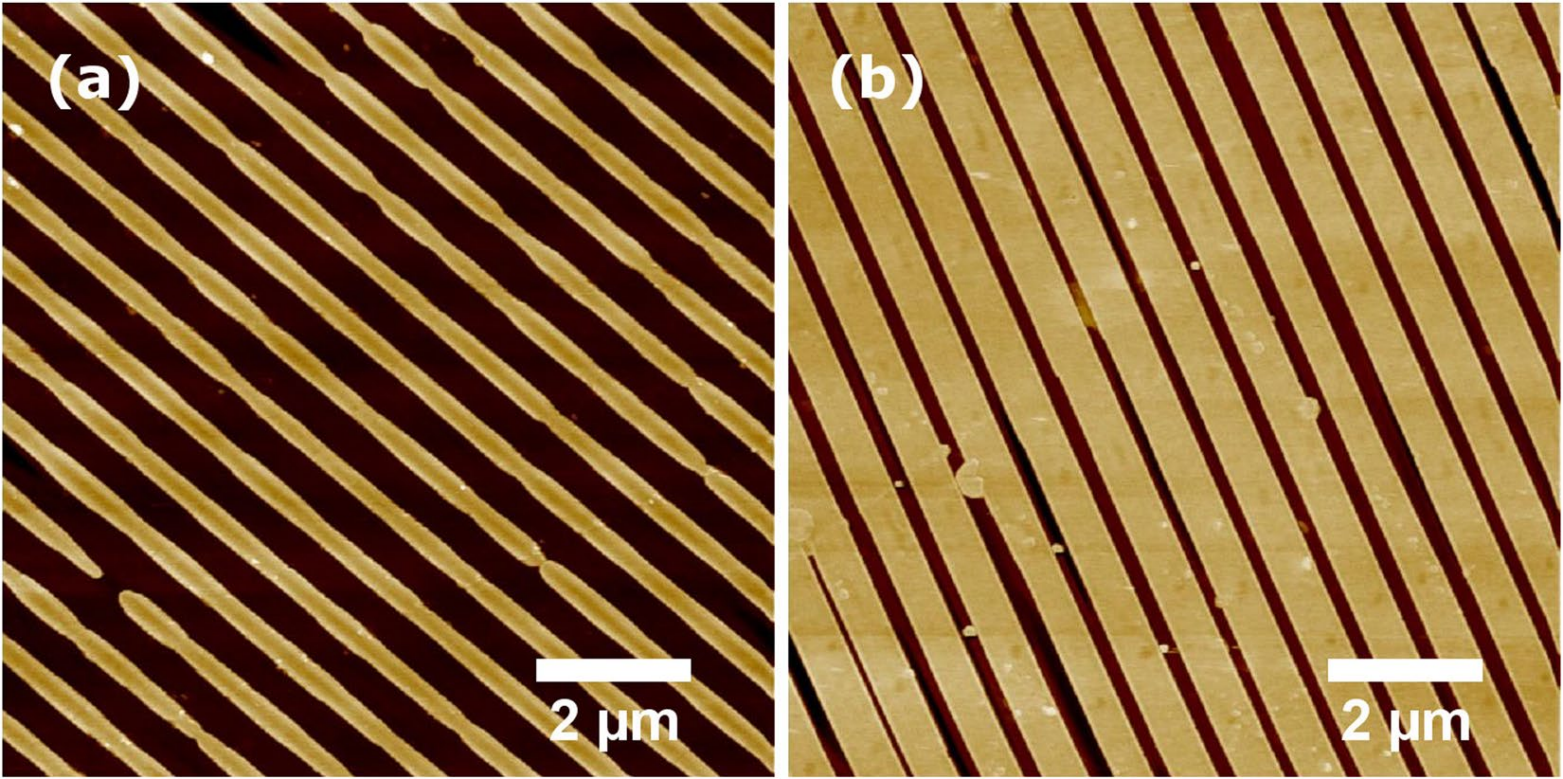
Sub-µm PPRKTP domain structure. Atomic force microscope image of the chemically etched (**a**) former patterned face and (**b**) former unpatterned face of a representative PPRKTP crystal.

RKTP is a very promising material for fabrication of fine-pitch domain structures. Its low Rb-doping (<0.3%) preserves the advantageous linear and nonlinear optical properties of regular undoped KTP, at the same time as this material shows an improved grey-tracking resistance^13^. Moreover, compared to undoped KTP, RKTP presents two-orders of magnitude lower ionic conductivity^14^, which results in reduced domain broadening and improved domain-growth along the z-direction. Although photolithographic patterning followed by electric field poling is the most developed and reliable periodic poling technique to date, it still remains challenging for sub-µm periods, since not only standard photolithography is unsuitable, but also methods to gain accurate control of the lateral domain growth are required. Therefore, in order to achieve reliable and consistent periodic poling we developed a technique based on coercive-field engineering^15^. First an aluminum grating mask with a period of 755 nm was created on one of the polar faces of RKTP crystals using an in-house built UV-laser interference lithography followed by liftoff process. The metal-stripe duty-cycle was 20%. Then, ion-exchange was performed through the periodic mask in order to obtain a coercive field grating in the crystals (see methods). The ion exchange has led to a coercive field increase of 1.5 kV/mm in the ion-exchanged regions. This increase is large enough to selectively induce domain-switching in the low-coercive field regions. Moreover, it allows for poling with planar electrodes, which alleviates the domain-broadening problem associated with the fringing fields using periodic metal electrodes^16^. After removing the Al grating, the samples were periodically poled by applying a single 5 ms-long electric-field pulse of triangular shape, which has proven beneficial for minimum domain broadening^7^. The peak magnitude of the electric pulse was adjusted between 5.5–6.5 kV/mm, depending on the specific ionic conductivity of each individual sample. All the periodically poled crystals showed excellent domain-grating uniformity over the entire aperture thickness (1 mm) and presented similar MOPO oscillation thresholds. The domain structure of one of the crystals can be seen in Fig. 3.

## Cascaded MOPO characterization

A PPRKTP with the QPM periodicity of 755 nm fabricated as described above was used to demonstrate cascaded counter-propagating nonlinear interactions. The crystal was pumped by a Ti:sapphire regenerative amplifier delivering 240 ps-long at full width at half maximum (FWHM) intensity stretched pulses at 1 kHz repetition rate. The pulses had a positive linear frequency chirp of 123 mrad/ps^2^, and a FWHM spectral bandwidth of 10 nm at the central wavelength of 800 nm. The pump light was polarized parallel to the crystal *z* axis, propagated along the *x*-axis, and was focused into the crystal to a beam radius (1/e^2^ intensity) of 105 µm. The pump pulse-energy was controlled through a half-wave plate and a polarizer arrangement (see Methods).

**Figure 4:**
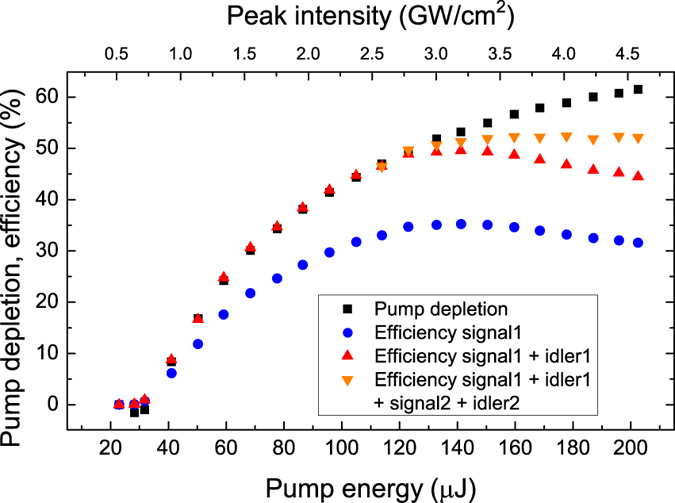
Conversion efficiencies and pump depletion. Pump depletion, *s1* efficiency and combined signal-and-idler efficiencies for the two MOPO cascade processes.

The threshold of the first MOPO process was reached for the pump energy of 39.8 µJ (corresponding to a peak intensity of 0.64 GW/cm^2^). Here the pump photons were split into co-propagating *s1* photons corresponding to the signal central wavelength of 1125 nm and counter-propagating *i1* idler photons with the central wavelength of 2763 nm. The measured MOPO efficiency and the pump depletion as a function of the pump energy are shown in Fig. 4. The threshold for the cascaded MOPO, in which *s1* serves as pump, was achieved at the *s1* energy of 22 µJ, and the corresponding primary pump energy was 108 µJ. The second MOPO process in the cascade generated a co-propagating signal, *s2*, and counter-propagating idler, *i2*, centered at 1898 nm and 2739 nm, respectively. At 202 µJ of the primary pump energy, the pump depletion was 61%; *s1* reached an energy of 62 µJ, achieving the total *s1* + *i1* power conversion efficiency of 43%. The cascaded signal *s2* energy was 9 µJ, with a *s2* + *i2* conversion efficiency of 8%. The maximum total conversion efficiency for the first MOPO process (*s1* + *i1*) was achieved at the pump energy of 130 µJ. At higher pump energies, the efficiency rolls-off, due to the depletion of *s1* by the second MOPO process (*s2* + *i2*). Note that when the pump energy reaches 150 µJ, there is an apparent discrepancy between pump depletion and total efficiency of the two MOPO processes. This can be attributed to a third cascaded process for which *s2* serves as a pump. In this case, the third MOPO generates a counter-propagating signal at 2762 nm and a co-propagating idler at 5.9 µm, which would be mostly absorbed in the material. The threshold of the first MOPO process can be used to estimate the effective second-order nonlinearity through the equation^6, 17^:

2 $${I}_{Pth}=\frac{{{\epsilon }}_{0}c{n}_{p}{n}_{i}{n}_{s}{\lambda }_{i}{\lambda }_{s}}{2{L}^{2}{d}_{eff}^{2}},$$

where ${\epsilon }_{0}$ is the permittivity of free space, *c* is the speed of light in vacuum, *L* is the interaction length, *d*_*eff*_ is the effective second-order nonlinearity, *n*_*k*_ is the refractive index for the corresponding waves ($k=p,i,s$ for pump, idler and signal, respectively) and *λ*_*k*_ is the wavelength. Taking into account that the length of the QPM grating was 6.4 mm, the estimated effective nonlinearity of our PPRKTP crystal is 9.77 pm/V. It should be noted that this value is close to the expected value (10.7 pm/V) for a perfect QPM grating in KTP^18^. The homogeneity of the domain-grating was assessed by measuring the conversion efficiency for *s1* in steps of 200 µm along the crystal z-axis at a pump energy well below the threshold of the second MOPO process. The crystal proved to be very homogeneous showing the *s1* efficiency of 18.2% with a standard deviation of 0.1% along the whole crystal thickness of 1 mm. To the best of our knowledge, this represents the most efficient and most homogenous sub-µm periodically poled crystal reported ever.

**Figure 5:**
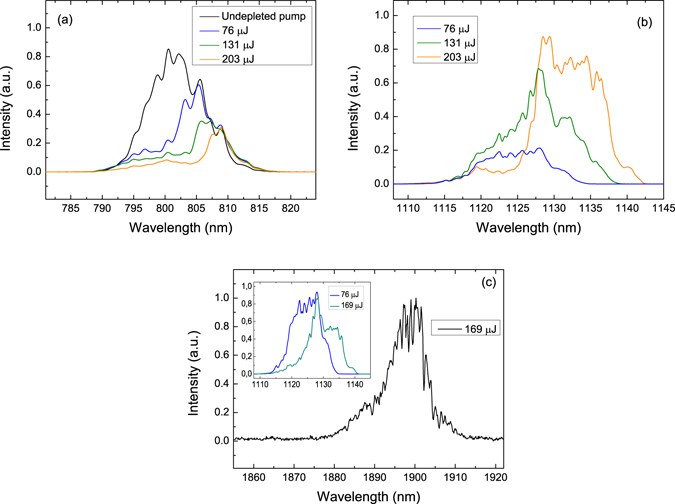
Pump and signal spectra. Spectra of (**a**) the pump, (**b**) *s1* and (**c**) *s2* at different pump energies, shown in the figures legends. Inset in (**c**) – Normalized *s1* spectra at different pump energies, showing *s1* depletion.

One of the unique features of the MOPO, resulting from the counter-propagating geometry of the interaction, is that the phase modulation of the pump is coherently transferred to the forward parametric wave, while the backward wave retains a narrow bandwidth^3, 19^. This implies that the positive frequency chirp of the pump will be transferred to *s1*, and, in turn, to *s2*. Figure 5 compares the spectra of (a) the pump, (b) *s1*, and (c) *s2* for different pump energies. Note that due to the linear chirp, the pump spectrum is not depleted uniformly. It occurs first on the short wavelength side of the pump spectrum, which here corresponds to the trailing edge of the pulse, reflecting the time delay required to establish distributed feedback. As the pump energy is increased, the time delay is reduced, and longer wavelengths in the pulse also get converted efficiently. For instance, at the pump energy of 203 µJ, the remaining undepleted part of the pump has FWHM length of 93 ps, which is comparable to the cumulative time of 80 ps that is required for the pump to travel through the QPM structure and the idler to travel back to the beginning of the structure, *L*(1/*v*_*gp*_ + 1/*v*_*gi*1_), where *v*_*g*_ denote the corresponding group velocities. At the pump energy of 79 µJ, when only one MOPO process is active, the generated *s1* pulse length can be estimated from the depleted part of the pump which has FWHM length of 141 ps. The measured FWHM frequency bandwidth of *s1* at this pump energy is 2.8 THz, which gives the *s1* chirp rate of 125 mrad/ps^2^, close to the chirp rate of 124.5 mrad/ps^2^ expected from the calculations based on the formulae in ref. 3.

At the pump energy of 131 µJ, the *s1* spectrum broadens to the low frequency side reflecting increased pump depletion at the lower frequency side of the spectrum. At the same time, at this pump energy the second MOPO process is already active and it starts depleting *s1* on the high-frequency side of the spectrum. This interplay of the two processes is even more clearly visible at the pump energy of 203 µJ, where the *s1* FWHM spectral width is 2.55 THz, i.e. actually narrower than the one obtained at the pump energy of 79 µJ. Here the energy from the *s1* high-frequency part of the spectrum with the corresponding phase modulation was transferred to the *s2* centered at 1897 nm. This depletion on the high-frequency side of the *s1* spectrum is clearly visible in the inset of the Fig. 5(c). The measured FWHM spectral bandwidth of the *s2* was 0.863 THz, which for the chirp transfer rate from *s1* to *s2*, $\partial {\omega }_{s2}/\partial {\omega }_{s1}$ of 1.005^3^, would give the *s2* FWHM pulse length of about 43 ps.

**Figure 6:**
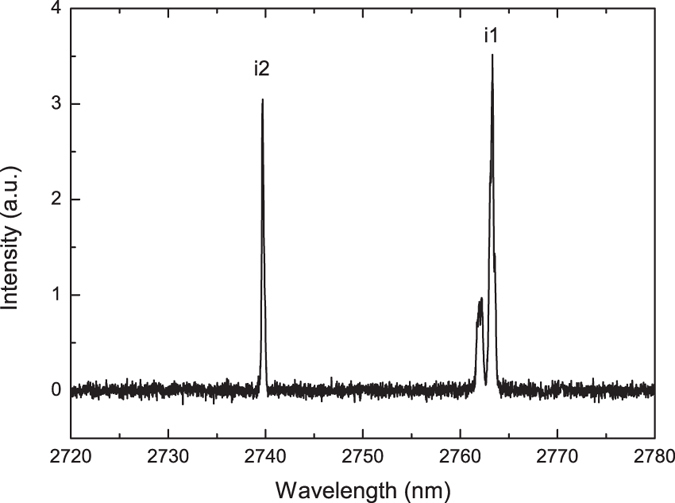
Idlers spectra. Spectra of the idlers waves, *i1* and *i2*, at a pump energy of 203 µJ.

The spectra of the backward-propagating waves generated in the cascaded MOPO processes measured at the pump energy of 203 µJ are shown in Fig. 6. The spectral resolution of the measurement was 0.1 nm, which corresponds to the frequency resolution of about 4 GHz in the spectral range of the idlers. As expected from the calculation results shown in Fig. 2 the wavelengths of the idlers generated in the two cascaded MOPO processes, *i1* and *i2*, change rather little as compared to the large steps in the central wavelengths of *s1* and *s2*. The idler wave *i1* is centered at 2763.3 nm, and *i2* is centered at 2739.7 nm. The measured FWHM spectral-width of *i1* and *i2* were 17 GHz and 10 GHz, respectively. From the MOPO signal- and idler-chirp ratios we can estimate the expected bandwidth. In the first MOPO process (*s1*, *i1*) of the cascade the theoretical ratio is 84, while for the second process (*s2*, *i2*), the ratio is substantially larger and equal to 201. This indicates that the bandwidth of the *i2* is expected to be narrower than that of *i1*, as indeed is the case. From the spectral bandwidth of the corresponding signals, we estimate the expected *i1* and *i2* bandwidths to be 33 GHz and 4.3 GHz, respectively. It should be noted however, that the pulse-length of the *s2*-*i2* pair of the second cascade is of the order of 40 ps, which would give the transform-limited bandwidth of about 10 GHz, in accordance with the measured value. The spectrum around *i1* at the same time consists of two clearly resolved peaks. The higher amplitude peak is indeed idler *i1* from the first MOPO process, while the other one with the FWHM of 28 GHz we attribute to the third MOPO cascade pumped by the *s2*, i.e. the signal from the second cascade. Calculations in Fig. 2 show that the third cascade is indeed expected in a PPRKTP with a 755 nm period, with a wavelength of 5.9 µm for the forward-propagating idler, *i3*, and a wavelength of 2.762 µm for the backward-propagating signal, *s3*. We could not observe the forward-propagating wave owing to large absorption of the material at this wavelength. The wavelength of the *s3* is on the other hand within the bandwidth of *i1* so we can expect that the third MOPO cascade is helped by cross-seeding from the idler of the first MOPO cascade. Another strong indication of the presence of the third cascade can be gleaned from the discrepancy of the measured pump depletion and the efficiency of the two first MOPO cascades at the pump energies above 140 µJ (see Fig. 4). We verified that this discrepancy is not caused by the polariton scattering^20^, once again attesting to the high quality of the QPM structure. Moreover, the idler spectrum below this pump energy does not contain the peak attributable to the third MOPO process.

## Discussion

The observation of the three MOPO cascades taking place very efficiently in the same QPM structure with sub-micrometer periodicity demonstrates high quality and homogeneity of the structures. The MOPO threshold even in these relatively short structures is low enough to consider applications where pumping with Q-switched lasers is employed. The domain-engineering technique presented could be potentially used to fabricate structures with larger domain aspect-ratios, either in terms of shorter periodicity or larger optical apertures. It is easy to predict that the MOPO can be scaled up in output energy to millijoules with the structuring techniques that we used in this work. This is appealing due to clear advantages of the MOPO as compared to the ordinary co-propagating optical parametric oscillators (OPOs). First, MOPO offers excellent control over the output spectrum both of the forward and the backward-propagating waves. Second, the upconversion of the signal and idler back to the pump is virtually absent in MOPO owing to the spatial separation of the intensity maxima of these waves. In contrast, the upconversion to the pump in OPOs is detrimental to the spatial and temporal coherence of the OPO output in most cases, and is limiting the efficiency of the device. Finally, MOPO is a much simpler and more robust device offering much lower sensitivity to variations of the crystal temperature or pump beam pointing direction.

The cascaded MOPO processes demonstrated in this work retain the temporal phase modulation over the cascade without the need of a separate reference wave to restrict the phase variations of the signal wave, as it would be necessary in co-propagating three-wave-mixing interactions. In MOPO, the backward-generated idler which is essentially determined by the fixed periodicity of the QPM grating serves as a built-in reference wave. Therefore, MOPO could make the frequency translation arrangements simpler and more robust. Moreover, the signal wavelengths in the MOPO cascade can be arranged by changing the wavelength of the primary pump. For instance, using the same PPRKTP structure with the periodicity of 755 nm, one can realize a cascade that ends up at degeneracy by pumping the structure at the wavelength of around 920 nm. The cascade with degenerate output can be used in realization of biphoton states and in self-referencing schemes for wavelength locking. A more colorful example is a cascade in the same QPM structure pumped at the wavelength of 532 nm. The cascade would produce a deep-red output at 654 nm and near-infrared output at 866 nm, which, in turn could be frequency doubled to generate deep-blue color at 433 nm. This might be perceived as similar to stimulated Raman cascade. However, the MOPO cascade has several important advantages: first, the frequency shift of the forward-propagating wave in the cascade can be designed and can be much larger than in any Raman medium; second, the “quantum defect” is not transferred to the phonon system ending up in heat generation, but is transferred to the backwards-propagating wave; third, due to the fact that MOPO is driven by the second-order nonlinear process, the threshold will always be lower than in stimulated Raman cascade devices.

## Methods

### Sample preparation

For our experiments we have used commercial, 1 mm thick, z-cut bulk Rb-doped KTP crystals. First a photoresist grating with a period of 755 nm was deposited on z^−^ faces of the crystals by in-house built UV interference lithography. Next, a 50 nm-thick Al film was evaporated over the grating, and the photoresist layer was lift-off. A planar ion-diffusion stop-layer was created on z^+^ faces by oxygen plasma etching. Next, the crystals were immersed into a molten nitrate salt bath, containing 20% KNO_3_, 73% RbNO_3_ and 7% Ba(NO_3_)_2_, at a temperature of 330 °C for 4 hours. Finally, the aluminum pattern was removed and the RKTP crystals were connected to the poling circuit using planar liquid electrodes. The periodically poled volume was approximately 6.4 × 3 × 1 mm^3^, along *x*, *y*, and *z* directions, respectively.

### Optical Experiments

The pump polarization and energy were controlled by a half-waveplate and polarizer arrangement. A small fraction of the pump light was reflected by a partial reflector (18% reflectivity) into a power meter in order to monitor the pump energy incident on the PPRKTP crystal. The z-polarized light was focused by a CaF_2_ lens (f = 200 mm) into the PPRKTP crystal to a *1/e* ^*2*^ intensity focal spot beam radius of 105 µm. The forward-propagating residual pump and signals beams were collimated with a BK7 lens (f = 200 mm) and launched into a BK7 prism in order to achieve spatial separation, while the backward-propagating idlers were separated from the pump using a dichroic mirror. Pump depletion was obtained by measuring the pump energy before the PPRKTP crystal and after the prism, using two calibrated power meters (Melles Griot 13PEM001). The transmission loss for the signals energies was obtained by measuring the pump energy loss while the pump propagated in the crystal away from the QPM grating. Using the same approach we measured the energies of the signals waves in order to obtain conversion efficiency, while the energies of the idler waves were determined using the Manley-Rowe relations.

The spectral measurements of the residual pump and *s1* waves were done using a fiber-coupled optical spectrum analyzer (OSA, ANDO AQ-6315A) with a 0.05 nm resolution. The *s2* spectrum was measured with a free-space imaging spectrometer (Jobin-Yvon iHR550), with a 0.14 nm resolution, whereas the spectra of the idlers were obtained with 0.1 nm-resolution using a fiber-coupled optical spectrum analyzer (Yokogawa AQ6376).
